# GluN2B and GluN2A-containing NMDAR are differentially involved in extinction memory destabilization and restabilization during reconsolidation

**DOI:** 10.1038/s41598-020-80674-7

**Published:** 2021-01-08

**Authors:** Andressa Radiske, Maria Carolina Gonzalez, Diana A. Nôga, Janine I. Rossato, Lia R. M. Bevilaqua, Martín Cammarota

**Affiliations:** 1 Memory Research Laboratory, Brain Institute, Federal University of Rio Grande do Norte, Av. Nascimento de Castro 2155, Natal, RN 59056-450 Brazil; 2 Edmond and Lily Safra International Institute of Neuroscience, Av. Alberto Santos Dumont 1560, Macaiba, RN 59280-000 Brazil; 3 Department of Physiology, Federal University of Rio Grande do Norte, Av. Sen. Salgado Filho 3000, Natal, RN 59064-741 Brazil

**Keywords:** Learning and memory, Extinction

## Abstract

Extinction memory destabilized by recall is restabilized through mTOR-dependent reconsolidation in the hippocampus, but the upstream pathways controlling these processes remain unknown. Hippocampal NMDARs drive local protein synthesis via mTOR signaling and may control active memory maintenance. We found that in adult male Wistar rats, intra dorsal-CA1 administration of the non-subunit selective NMDAR antagonist AP5 or of the GluN2A subunit-containing NMDAR antagonist TCN201 after step down inhibitory avoidance (SDIA) extinction memory recall impaired extinction memory retention and caused SDIA memory recovery. On the contrary, pre-recall administration of AP5 or of the GluN2B subunit-containing NMDAR antagonist RO25-6981 had no effect on extinction memory recall or retention per se but hindered the recovery of the avoidance response induced by post-recall intra-CA1 infusion of the mTOR inhibitor rapamycin. Our results indicate that GluN2B-containing NMDARs are necessary for extinction memory destabilization whereas GluN2A-containing NMDARs are involved in its restabilization, and suggest that pharmacological modulation of the relative activation state of these receptor subtypes around the moment of extinction memory recall may regulate the dominance of extinction memory over the original memory trace.

## Introduction

Recall reactivates memories that lay dormant and may affect their strength and endurance. When triggered by a single brief re-presentation of the conditioned stimulus in the absence of the unconditioned stimulus, recall can destabilize well-consolidated memories, which must then undergo protein synthesis-dependent reconsolidation to persist. Conversely, repetitive non-reinforced recall events may induce extinction, a protein synthesis-dependent process that generates a new memory that prevents the original one from continuing to control behavior. Notably, extinction memory may also re-enter an instability phase upon recall and must be reconsolidated to maintain its dominance over the extinguished original trace^1,2^. In the case of fear-motivated avoidance, the reconsolidation of extinction memory requires mTOR-dependent BDNF expression in the dorsal hippocampus^3,4^, but the upstream pathways controlling this process remain largely unknown.

N-methyl-D-aspartic acid receptors (NMDARs) are heterotetrameric ionotropic receptors formed by co-assembly of seven subunits (GluN1, GluN2A-D, and GluN3A-B) that mediate a Ca^2+^-permeable component of glutamatergic neurotransmission. Most native NMDARs contain two obligatory GluN1 subunits and two GluN2 subunits, which confer distinctive channel, ligand-binding and signaling properties to NMDAR subtypes and enable them to fulfill specific physiological functions. In particular, GluN2A- and GluN2B-containing NMDARs control bidirectional synaptic plasticity^5^, play key roles in memory consolidation and extinction^6,7^, and underlie the destabilization and restabilization of different memory types during reconsolidation^8–13^. Here, we analyzed whether hippocampal NMDARs are necessary for extinction memory reconsolidation by assessing the effect of the intra-dorsal CA1 administration of non-subunit specific and subunit-specific NMDAR antagonists at different time points around the moment of step-down inhibitory avoidance (SDIA) extinction memory recall.

## Results

### Post-recall intra-CA1 administration of the non-subunit specific NMDAR antagonist AP5 hinders SDIA extinction memory and induces SDIA memory renewal

**Figure 1:**
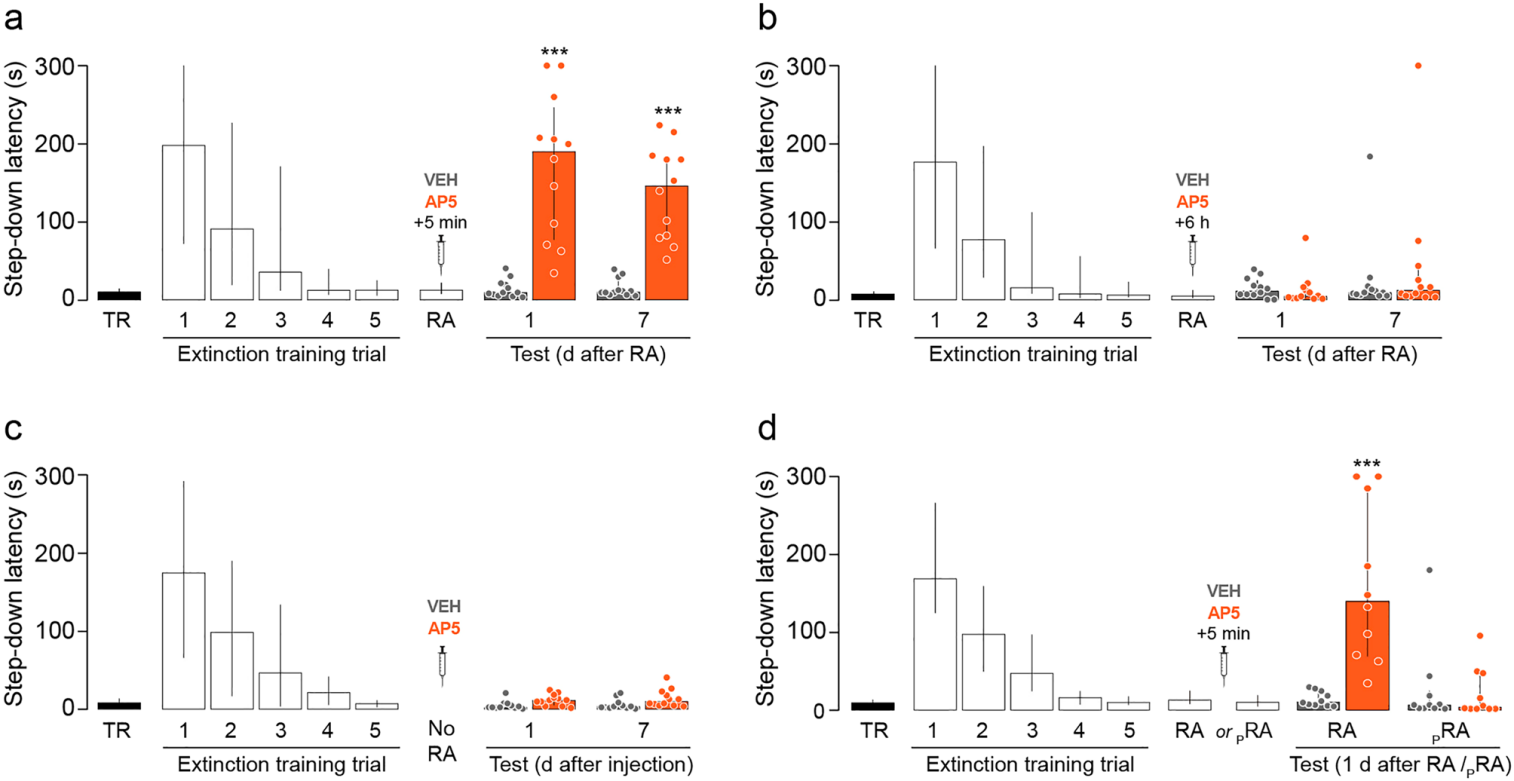
Post-recall NMDAR blockade hinders SDIA extinction memory and induces SDIA memory recovery. (**a**) Animals were trained in SDIA (TR; 0.4 mA/2 s) and beginning 24 h later they were submitted to one daily extinction training trial for 5 consecutive days. Twenty-four hours after the last extinction training trial, SDIA extinction memory was reactivated (RA) and, 5 min thereafter, the animals received bilateral intra-dorsal CA1 infusions of vehicle (VEH; 1% DMSO in saline) or the NMDAR antagonist AP5 (5 µg/side). Retention was assessed 1 day and 7 days later (Test). (**b**) Animals were treated as in A except that they received intra-CA1 infusions of VEH or AP5 6 h after RA. (**c**) Animals were treated as in A, except that RA was omitted (No RA). (**d**) Animals were treated as in A, but a group of them received VEH or AP5 5 min after a pseudo-reactivation extinction session (pRA) carried out in an SDIA training box modified to be non-aversive (NA) for SDIA-trained animals (test latency in SDIA: Median = 162 s; IQR = 74–244.5 s; test latency in NA: Median: 10 s; IQR = 7.5–17.5 s; U = 3.00, *p* = 0.0003, SDIA vs NA in Mann–Whitney test). The non-aversive box was similar in dimensions to the SDIA-training apparatus but was painted gray and the elevated platform was made of transparent plexiglass instead of wood. Data are expressed as median ± IQR. (***) *p* < 0.001 versus VEH in Mann–Whitney test; n = 10–12 animals per group.

To study the role of hippocampal NMDARs in the reconsolidation of extinction memory, we first trained adult male Wistar rats in one-trial step-down inhibitory avoidance (SDIA; 0.4 mA/2 s foot shock), a learning task that induces a long-lasting hippocampus dependent fear-motivated avoidance memory^14,15^ and then, beginning one day post-training, we re-exposed the animals to the SDIA training apparatus in the absence of the foot shock once daily for 5 consecutive days. This procedure generates a hippocampus dependent SDIA extinction memory^16–18^ resistant to spontaneous recovery, renewal and reinstatement^1,3^. Twenty four hours after the last extinction training trial, we submitted the animals to an extinction memory reactivation session (RA), and 5 min or 6 h later they received bilateral intra-dorsal CA1 infusions of vehicle (VEH; 1% DMSO in saline) or the non-subunit specific NMDAR antagonist D(-)-2-Amino-5-phosphonopentanoic acid (AP5; 5 µg/side). Retention was evaluated twice, 1 day and 7 days after RA. We found that AP5 impaired SDIA extinction memory retention and recovered the SDIA response when injected 5 min but not 6 h after RA (Fig. 1a,b, 1 day after RA: U = 1, *p* < 0.0001, VEH vs AP5; 7 days after RA: U = 0, *p* < 0.0001, VEH vs AP5 5 min after RA in Mann–Whitney test). AP5 did not affect SDIA extinction memory when given 24 h after the last extinction training trial in the absence of RA (Fig. 1c) or when administered 5 min after a pseudo-RA session carried out in a non-aversive training box (Fig. 1d, RA: U = 0, *p* < 0.0001, VEH vs AP5; pRA: U = 42.50, *p* = 0.5875, VEH vs AP5 in Mann–Whitney test).

### Pre-recall intra-CA1 administration of the non-subunit specific NMDAR antagonist AP5 does not affect SDIA extinction memory expression or retention but hampers the amnesic effect of reconsolidation blockers

**Figure 2:**
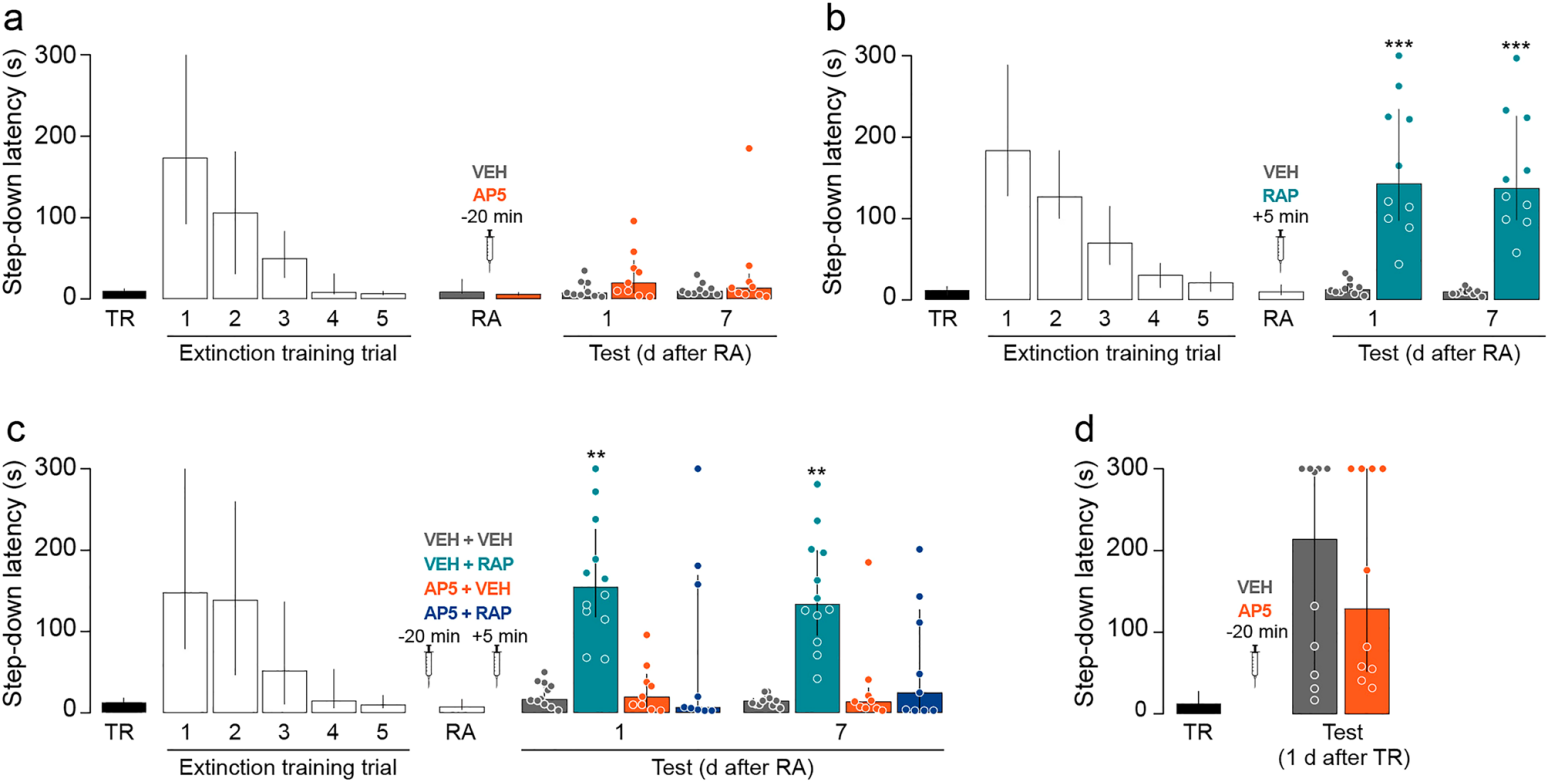
Pre-recall NMDAR blockade does not affect SDIA extinction memory but impedes the amnesic effect of reconsolidation inhibition. (**a**) Animals were trained in SDIA (TR; 0.4 mA/2 s) and beginning 24 h later they were submitted to one daily extinction training trial for 5 consecutive days. One day after the last extinction training trial, animals received bilateral intra-CA1 infusions of vehicle (VEH; 1% DMSO in saline) or the NMDAR antagonist AP5 (5 µg/side), and 20 min thereafter, SDIA extinction memory was reactivated (RA). Retention was assessed 1 day and 7 days later (Test). (**b**) Animals were treated as in A except that they received intra-CA1 infusions of VEH or rapamycin (RAP; 0.02 µg/side), an inhibitor of mammalian target of RAP (mTOR) 5 min after RA. (**c**) Animals were treated as in A and ,5 min after RA, they received bilateral intra-dorsal CA1 infusions of VEH or RAP. (**d**) Animals trained in SDIA received bilateral intra-CA1 infusions of VEH or AP5 one day post-training and, 20 min later, were submitted to a SDIA memory retention test. Data are expressed as median ± IQR. (**) *p* < 0.01, (***) *p* < 0.001 versus VEH in Dunn's multiple comparisons after Kruskal–Wallis test; n = 9–12 animals per group.

To evaluate the effect of pre-recall hippocampal NMDAR inhibition on SDIA extinction memory, we submitted SDIA-trained animals to the extinction protocol described above. Twenty-four hours after the last extinction training trial the animals received bilateral intra-dorsal CA1 infusions of VEH or AP5 and 20 min later were submitted to RA. We found that pre-RA AP5 did not affect SDIA extinction memory recall or retention (Fig. 2a) but impeded the recovery of avoidance induced by post-RA intra-CA1 administration of rapamycin (RAP; 0.02 µg/side), an inhibitor of mammalian target of RAP (mTOR), a kinase that regulates synaptic protein synthesis through the phosphorylation of eukaryotic initiation factor 4E-binding protein 1 and p70 ribosomal S6 kinase^19^ and is required for SDIA extinction memory reconsolidation in the hippocampus^4^ (Fig. 2b, 1 day after RA: U = 0, *p* < 0.0001, VEH vs RAP; 7 days after RA: U = 0, *p* < 0.0001, VEH vs RAP in Mann–Whitney test; Fig. 2c, 1 day after RA: H = 17.73, *p* = 0.0005; *p* < 0.01 for VEH + VEH vs VEH + RAP, *p* < 0.01 for VEH + RAP vs AP5 + VEH, *p* < 0.05 for VEH + RAP vs AP5 + RAP; 7 days after RA: H = 17.74, *p* = 0.0005; *p* < 0.01 for VEH + VEH vs VEH + RAP, *p* < 0.01 for VEH + RAP vs AP5 + VEH, *p* < 0.05 for VEH + RAP vs AP5 + RAP in Dunn's multiple comparisons after Kruskal–Wallis test). At the dose used in our experiments, AP5 did not affect SDIA memory recall (Fig. 2d).

### Hippocampal GluN2B-containing NMDARs mediate SDIA extinction memory destabilization during recall whereas GluN2A-containing NMDARs are necessary for SDIA extinction memory reconsolidation

**Figure 3:**
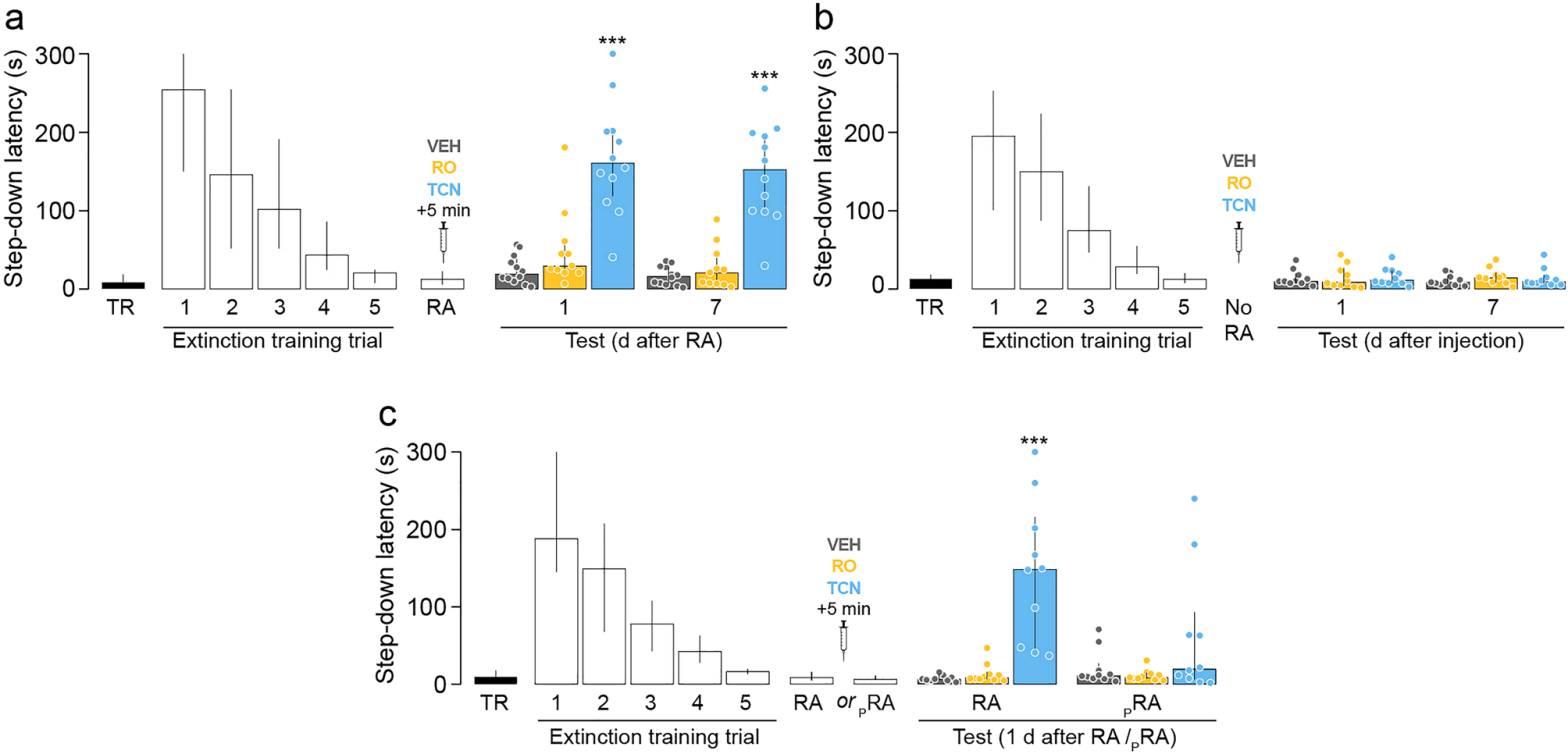
GluN2A-NMDARs are required for SDIA extinction memory restabilization. (**a**) Animals were trained in SDIA (TR; 0.4 mA/2 s) and, beginning 24 h later, they were submitted to one daily extinction training trial for 5 consecutive days. Twenty-four hours after the last extinction training trial, SDIA extinction memory was reactivated (RA) and, 5 min thereafter, the animals received bilateral intra-dorsal CA1 infusions of vehicle (VEH; 0.1% DMSO in saline), the GluN2B-containing NMDAR antagonist RO25-6981 (RO; 2.5 µg/side), or the GluN2A-containing NMDAR antagonist TCN201 (TCN; 0.05 µg/side). Retention was assessed 1 day and 7 days later (Test). (**b**) Animals were treated as in A except that RA was omitted (No RA). (**c**) Animals were treated as in A, but a group of them received bilateral intra-CA1 infusions of VEH, RO or TCN 5 min after a pseudo-reactivation extinction session (pRA) carried out in an SDIA training box modified to be non-aversive (NA) for SDIA-trained animals. The non-aversive box was similar in dimensions to the SDIA-training apparatus but was painted gray and the elevated platform was made of transparent plexiglass instead of wood. Data are expressed as median ± IQR. (***) *p* < 0.001 versus VEH in Dunn's multiple comparisons after Kruskal–Wallis test; n = 10–12 animals per group.

**Figure 4:**
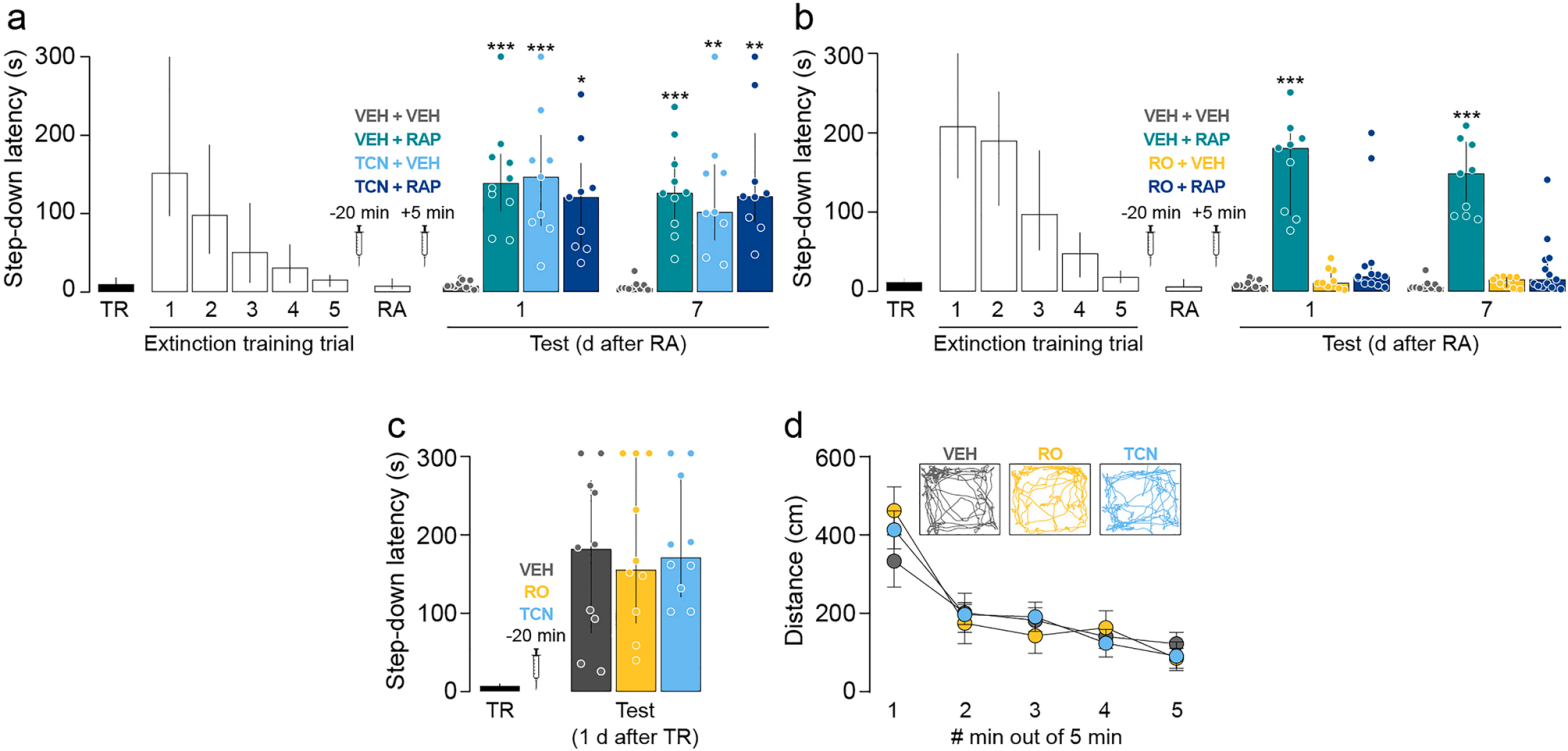
GluN2B-NMDARs are required for SDIA extinction memory destabilization. (**a**) Animals were trained in SDIA (TR; 0.4 mA/2 s) and, beginning 24 h later, they were submitted to one daily extinction training trial for 5 consecutive days. One day after the last extinction training trial, animals received bilateral intra-CA1 infusions of vehicle (VEH; 0.1% DMSO in saline) or the GluN2A-containing NMDAR antagonist TCN201 (TCN; 0.05 µg/side), and 20 min later, SDIA extinction memory was reactivated (RA). Five minutes after RA, rats received bilateral intra-dorsal CA1 infusions of VEH or rapamycin (RAP; 0.02 µg/side), an inhibitor of mammalian target of RAP (mTOR). Retention was assessed 1 day and 7 days later (Test). (**b**) Animals were treated as in A except that 20 min before RA they received bilateral intra-CA1 infusions of VEH or the GluN2B-containing NMDAR antagonist RO25-6981 (RO; 2.5 µg/side). (**c**) Animals trained in SDIA received bilateral intra-CA1 infusions of VEH, RO or TCN one day post-training and, 20 min later, were submitted to a SDIA memory retention test. (**d**) Animals received bilateral intra-dorsal CA1 infusions of VEH, TCN or RO and 20 min later were submitted to a 5 min-long open field arena exploration session to determine locomotor activity. Representative traces show locomotor activity from rats that received VEH, TCN or RO. Data are expressed as median ± IQR or mean ± SEM. (*) *p* < 0.05, (**) *p* < 0.01, (***) *p* < 0.001 versus VEH in Dunn's multiple comparisons after Kruskal–Wallis test; n = 9–13 animals per group.

GluN2A-containing and GluN2B-containing NMDAR regulate different cellular events in the amygdala during fear memory reconsolidation, controlling restabilization and destabilization phases, respectively^13,20^. Therefore, because our results with AP5 are consistent with a dual role of hippocampal NMDAR in extinction memory reconsolidation, we analyzed whether GluN2A-containing NMDAR and GluN2B-containing NMDAR also mediate SDIA extinction memory restabilization and destabilization differentially. To evaluate the involvement of these two NMDAR subtypes in extinction memory restabilization, we submitted SDIA-trained animals to the SDIA extinction protocol, as described above. One day after the last extinction training session, SDIA extinction memory was reactivated and 5 min later the animals received bilateral intra-dorsal CA1 infusions of VEH, the GluN2B-containing NMDAR antagonist RO25-6981 (RO; 2.5 µg/side), or the GluN2A-containing NMDAR antagonist TCN201 (TCN; 0.05 µg/side). As can be seen in Fig. 3, TCN, but not RO, impaired extinction memory retention and induced SDIA memory recovery 1 day and 7 days post-RA (Fig. 3a, 1 day after RA: H = 20.10, *p* < 0.0001; *p* < 0.001 for VEH vs TCN, *p* < 0.01 for RO vs TCN; 7 days after RA: H = 21.51, *p* < 0.0001; *p* < 0.001 for VEH vs TCN, *p* < 0.001 for RO vs TCN in Dunn's multiple comparisons after Kruskal–Wallis test). Neither RO nor TCN affected extinction memory retention when administered 24 h after the last extinction training trial in the absence of RA (Fig. 3b) or when given 5 min after a pseudo-RA session carried out in a non-aversive training box (Fig. 3c, RA: H = 19.30, *p* < 0.0001; *p* < 0.001 for VEH vs TCN, *p* < 0.01 for RO vs TCN; pRA: H = 2.006, *p* = 0.3667 in Dunn's multiple comparisons after Kruskal–Wallis test). We next evaluated whether GluN2B and GluN2A-containing NMDAR mediate SDIA extinction memory destabilization. To that end, SDIA-trained animals were submitted to the SDIA extinction protocol and 24 h after the last extinction training trial received bilateral intra-dorsal CA1 infusions of VEH, RO or TCN. SDIA extinction memory was reactivated 20 min after the injections and 5 min thereafter the animals received VEH or RAP in dorsal CA1. Retention was assessed 24 h post-RA. TCN did not affect extinction memory recall 20 min post-injection but TCN, RAP and TCN + RAP impaired SDIA extinction memory retention 1 day and 7 days after RA, causing the reappearance of the SDIA response (Fig. 4a, 1 day after RA: H = 20.65, *p* = 0.0001; *p* < 0.001 for VEH + VEH vs VEH + RAP, *p* < 0.001 for VEH + VEH vs TCN + VEH, *p* < 0.05 for VEH + VEH vs TCN + RAP; 7 days after RA: H = 19.96, *p* = 0.0002; *p* < 0.001 for VEH + VEH vs VEH + RAP, *p* < 0.01 for VEH + VEH vs TCN + VEH, *p* < 0.01 for VEH + VEH vs TCN + RAP in Dunn's multiple comparisons after Kruskal–Wallis test). RO did not affect extinction memory recall 20 min post-injection either but had no effect per se on retention and blocked the recovery of avoidance induced by post-RA RAP administration (Fig. 4b, 1 day after RA: H = 21.18, *p* < 0.0001; *p* < 0.001 for VEH + VEH vs VEH + RAP, *p* < 0.01 for VEH + RAP vs RO + VEH, *p* < 0.05 for VEH + RAP vs RO + RAP; 7 days after RA: H = 22.77, *p* < 0.0001; *p* < 0.001 for VEH + VEH vs VEH + RAP, *p* < 0.01 for VEH + RAP vs RO + VEH, *p* < 0.05 for VEH + RAP vs RO + RAP in Dunn's multiple comparisons after Kruskal–Wallis test). Neither RO nor TCN affected SDIA memory recall or locomotor activity when given in dorsal CA1 20 min before a SDIA memory retention test (Fig. 4c) or a 5 min-long free-exploration session in an open-field arena, respectively (Fig. 4d; F(8, 92) = 1.710, *p* = 0.1065 for interaction; F(2, 23) = 0.02170, *p* = 0.9786 for treatment effect; F(4, 92) = 50.11, *p* < 0.0001 for time effect in two-way RM ANOVA).

## Discussion

Our findings confirm that extinction learning does not erase SDIA memory but creates a new trace that competes with it for controlling behavior, and corroborate that SDIA extinction memory enters an instability phase when recalled and must be restabilized through reconsolidation to maintain the learned avoidance response inhibited. Importantly, our data demonstrate that SDIA extinction memory destabilization and restabilization necessitate hippocampal NMDARs activation and indicate that GluN2B- and GluN2A-containing NMDAR subtypes, respectively, are differentially involved in these processes. This assertion is based on experiments showing that post-recall infusion of AP5 and TCN, but not of RO, impaired extinction memory retention lastingly and induced the recovery of avoidance in a time-dependent manner whereas pre-recall administration of AP5 and RO, but not of TCN, rendered the reactivated extinction memory trace resistant to the amnesia caused by mTOR inhibition. Our conclusions are further supported by the fact that neither AP5 nor TCN affected extinction memory when its reactivation was omitted or when they were given after exploration of a non-aversive environment, as well as by results showing that pre-recall infusion of AP5, TCN and RO had no effect on SDIA memory expression. Moreover, our data agree with reports that pre-recall administration of non-subunit selective or GluN2B-selective NMDAR antagonists prevents contextual fear memory updating^10,21^ and others showing that destabilized memories require GluN2A-containing NMDAR upregulation to regain stability and persist^13,22,23^. Milton and coworkers^13^ attributed the differential involvement of GluN2B and GluN2A-containing NMDARs in the destabilization and restabilization of reconsolidating memories to the fact that GluN2B-NMDARs regulate the activation state of the protein degradation system that modulates memory lability^24^ whereas GluN2A-NMDARs promote CREB phosphorylation^25^ and long-term potentiation^26^, which are associated with memory re-encoding and maintenance^27–29^.

Psychotherapeutic interventions based on memory extinction are initially effective in reducing the exacerbated recall of traumatic events that afflicts post-traumatic stress disorder (PTSD) patients. However, PTSD symptoms usually return spontaneously, or due to unexpected triggers, after the end of psychotherapy. Therefore, most research on the psychopharmacology of extinction has revolved around the search for tools to improve extinction learning, based on the premise that this could prevent fear recovery. Because it has been repeatedly shown that NMDAR blockade impairs extinction retention whereas NMDAR function enhancement facilitates fear extinction in experimental animals^30–33^, NMDARs have taken center stage in this enterprise. In this regard, it has been reported that the NMDAR partial agonist d-cycloserine (DCS) increases exposure therapy effects^34–36^, although the available data are inconclusive^37,38^ and experimental animals as well as humans treated with DCS during extinction learning show substantial recovery of learned fear, suggesting that DCS may not prevent PTSD relapse^39–42^. These studies, together with several others, suggest that extinction can indeed be modulated by drugs, but not lastingly^43–45^. Our findings that SDIA memory is still able to assume control of behavior after undergoing an extinction procedure that generates an extinction memory resistant to spontaneous recovery, renewal and reinstatement but sensitive to recall-induced GluN2B-containing NMDAR-dependent destabilization indicate that it is this destabilization what enables the reappearance of avoidance, and lead us to propose that blockers of these receptors might be suitable tools to prevent PTSD relapse. It is crucial to emphasize here that memory destabilization can result not only from explicit recall, but also from exposure to subtle signals and cues unable to elicit discernible behavioral responses^46^. In addition, it is important to consider that because of local animal housing regulations and facility constraints our experiments were carried out exclusively on adult male rats although sex and age related differences in NMDAR endogenous modulation and subunit composition have been reported^47–49^, women are twice as likely to develop PTSD than men^50,51^, and PTSD management in children might require a differential therapeutic approach^52,53^.

## Methods

### Animals

We used 3-month-old male Wistar rats, weighing 300–350 g at the start of the experiments. They were housed in groups of 5 with free access to water and food in a holding room maintained at 22–23 °C on a normal light cycle (12 h light:12 h dark; lights on at 6.00 A.M.). Experiments were performed during the light phase of the cycle. All procedures were performed in accordance with the USA National Institutes of Health Guidelines and Regulations for Animal Care and were approved by the local institutional ethics committee (Comissão de Ética no Uso de Animais—CEUA, Federal University of Rio Grande do Norte).

### Surgery and drug infusion procedures

Animals were submitted to stereotaxic surgery and implanted with 22-gauge guides aimed at the CA1 region of the dorsal hippocampus (coordinates in mm: anteroposterior, − 4.2; laterolateral, ± 3.0; dorsoventral, − 3.0). Experiments began 1 week after surgery. At the time of drug delivery, infusion cannulas were fitted into the guides and injections (1 µl/side) performed during 60 s with a microinjection pump. Cannulas were left in place for 60 additional seconds to minimize backflow. Cannula placement was verified postmortem. To do that, 2–4 h after the last behavioral test we infused 1 μl of 4% methylene-blue as described above; the extension of the dye 30 min thereafter was taken as an indicator of drug diffusion. Only data from animals with correct implants were analyzed (see Supplementary Fig. S1).

### Step-down inhibitory avoidance (SDIA) training

The SDIA training box (50 × 25 × 25 cm) consisted of a Plexiglas chamber with a grid floor through which a scrambled electric shock could be delivered to the rat's feet and a wooden platform (5 × 8 × 25 cm) at the left end of the grid floor. For training (TR), each animal was placed on the platform and when it stepped down and placed its four paws on the grid, received a mild foot shock (0.4 mA/2 s). Immediately thereafter the rat was withdrawn from the training box.

### SDIA memory extinction protocol

To extinguish SDIA memory, animals were submitted to five daily extinction sessions during which they were placed on the training box platform and allowed to freely explore the apparatus for 30 s after stepping down to the grid. To reactivate the SDIA extinction memory (RA), the animals were placed on the platform and after stepping down from it were immediately removed from the training box.

### Drugs

D( −)-2-Amino-5-phosphonopentanoic acid (AP5; 5 µg/side), RO 25–6981 (RO; 2.5 µg/side), TCN 201 (TCN; 0.05 µg/side) and rapamycin (RAP; 0.02 µg/side) were dissolved according to the manufacturer's instructions and stored protected from light at − 20 °C until use. Aliquots were thawed and diluted to working concentration in 1% DMSO in sterile saline (pH 7.2) on the day of the experiment. The doses used were determined based on pilot experiments and previous studies showing the behavioral and biochemical effects of each compound^3,4,54,55^. An equal volume of 1% DMSO in sterile saline served as vehicle control.

### Data analysis

Statistical analyses were performed using GraphPad Prism 8 software. Significance was set at *p* < 0.05. A 300 s ceiling was imposed on retention test session latency. Data were analyzed using two-tailed Mann–Whitney U test, Kruskal–Wallis test followed by Dunn's post hoc comparisons, or two-way RM ANOVA followed by Bonferroni's multiple-comparisons test.

## Supplementary information

Supplementary information.

## Data Availability

Data are available upon request by contacting the corresponding author.
