# Supplementary material for: GluN2B and GluN2A-containing NMDAR are differentially involved in extinction memory destabilization and restabilization during reconsolidation
Source: Sci Rep. 2021 Jan 8;11:186. doi: 10.1038/s41598-020-80674-7 (PMC7794413; doi:10.1038/s41598-020-80674-7)
Supplement: Supplementary file 1 — Supplementary information. [file 41598_2020_80674_MOESM1_ESM.docx]

**GluN2B and GluN2A-containing NMDAR are differentially involved in extinction memory destabilization and restabilization during reconsolidation**

Andressa Radiske^1^, Maria Carolina Gonzalez^1,2^, Diana A. Nôga^1^, Janine I. Rossato^1,3^, Lia R. M. Bevilaqua^1^, and Martín Cammarota^1^

^1^Memory Research Laboratory, Brain Institute, Federal University of Rio Grande do Norte, Av. Nascimento de Castro 2155, RN 59056-450, Natal, Brazil. ^2^Edmond and Lily Safra International Institute of Neuroscience, Av. Alberto Santos Dumont 1560, RN 59280-000, Macaiba, Brazil. ^3^Departament of Physiology, Federal University of Rio Grande do Norte, Av. Sen. Salgado Filho 3000, RN 59064-741, Natal, Brazil. *****Correspondence should be addressed to Martín Cammarota at martin.cammarota@neuro.ufrn.br.


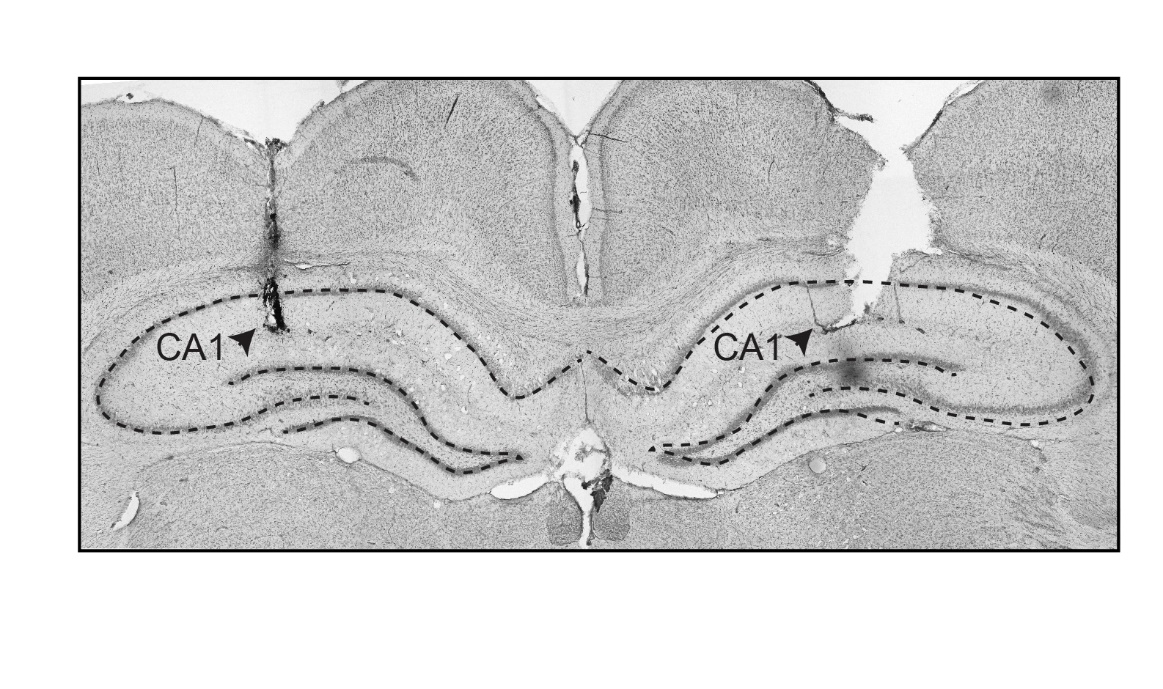


**Supplementary Figure S1.** Representative photomicrograph of Nissl-stained section showing infusion cannula track terminating in the CA1 region of the dorsal hippocampus.
